# Beyond Symptom Control: Remission as a Depth-Stratified Treatment Goal in Severe Asthma

**DOI:** 10.3390/jcm15197686

**Published:** 2026-10-04

**Authors:** Martina Votto, Fabio Manzo, Mauro Maniscalco, Giorgio Castellana, Giuseppe Cascone, Agostino Buonauro, Antimo Papa, Roberto Formisano, Christian Basile, Guido Grassi, Pasquale Ambrosino

**Affiliations:** 1 Maternity and Paediatric Department, Azienda Sanitaria Locale, 82100 Benevento, Italy; 2 Fleming Clinical Laboratory, 81020 Casapulla, Italy; fabioce26@gmail.com; 3 Istituti Clinici Scientifici Maugeri IRCCS, Pulmonary Rehabilitation Unit of Telese Terme Institute, 82037 Telese Terme, Italy; mauro.maniscalco@icsmaugeri.it; 4 Department of Advanced Biomedical Science, Federico II University, 80131 Naples, Italy; 5 Istituti Clinici Scientifici Maugeri IRCCS, Pulmonary Rehabilitation Unit of Bari Institute, 70124 Bari, Italy; giorgio.castellana@icsmaugeri.it; 6 Centro Medicina del Lavoro Vesuvio, 80035 Nola, Italy; giuseppecascone@cmlvesuvio.com; 7 Cardiology Unit, “Santa Maria della Pietà” Hospital, Local Health Authority Napoli 3 Sud (ASL Napoli 3 Sud), 80035 Nola, Italy; stino84@gmail.com; 8 Istituti Clinici Scientifici Maugeri IRCCS, Cardiac Rehabilitation Unit of Telese Terme Institute, 82037 Telese Terme, Italy; antimo.papa@icsmaugeri.it (A.P.); roberto.formisano@icsmaugeri.it (R.F.); 9 Department of Clinical Science and Education, Karolinska Institutet, 171 77 Stockholm, Sweden; christian.basile.94@gmail.com; 10 ANMCO Research Center, Heart Care Foundation, 50121 Florence, Italy; 11 Department of Medicine and Surgery, University of Milano-Bicocca, 20162 Milano, Italy; guido.grassi@unimib.it; 12 Istituti Clinici Scientifici Maugeri IRCCS, Medical Directorate of Telese Terme Institute, 82037 Telese Terme, Italy

**Keywords:** type 2 inflammation, severe asthma, chronic rhinosinusitis with nasal polyps, remission, biologic therapy, treat-to-target, airway remodeling, rehabilitation, exercise, disability

## Abstract

The management of severe asthma has shifted from symptom control to the ambition of remission, a goal borrowed from rheumatology. Biologics targeting immunoglobulin E, interleukin-5 and its receptor, the interleukin-4 receptor alpha subunit, and thymic stromal lymphopoietin have made sustained quiescence realistic for many patients, yet estimates that roughly one-third achieve remission shift by tens of percentage points depending on the definition used. This narrative review advances two linked arguments. First, that the heterogeneity of definitions, not any shortage of effective agents, is the central obstacle to using remission as a treatment target. Second, that remission is best read as a multidimensional construct in which clinical, functional and biological quiescence are distinct axes, reached independently and often discordantly, with complete remission denoting their convergence rather than the last step of a sequence. Where each biologic acts within the type 2 cascade determines which biomarkers move under treatment and, only hypothetically, how deep remission can reach. Structural remodeling and the fact that current agents suppress rather than reset inflammation cap that depth, so remission remains a pharmacologically maintained state on treatment. Concomitant chronic rhinosinusitis with nasal polyps is treated as a modifier of asthma remission rather than a separate endpoint. The framework is conceptual and unvalidated. The priority is a harmonized, depth-resolved definition, explicit about treatment burden and patient-centered outcomes, against which any therapy can be judged.

## 1. Introduction

For most of modern asthma history, the therapeutic contract offered to patients was modest, focusing on symptom relief and suppression of exacerbations, pursued with corticosteroids and, when these failed, with additional controllers. Control, not cure, was the horizon of expectation, and it was accepted that the disease would relapse whenever treatment was withdrawn. That contract has been rewritten. Recognition that a large share of severe airway disease is driven by type 2 (T2) inflammation [1], and the arrival of monoclonal antibodies that can interrupt that inflammation at defined points, has shifted the conversation from whether symptoms can be contained to whether the disease itself can be brought to a standstill [2]. The vocabulary has shifted with it, and the word now used to name the goal, remission, has been deliberately borrowed from rheumatology, where a treat-to-target philosophy built around defined states of low or absent disease activity transformed the management of rheumatoid arthritis a generation ago [2].

The borrowing is more than rhetorical. Although long-standing estimates place the proportion of asthma that remains severe despite optimized inhaled therapy between five and ten percent, more rigorous application of consensus criteria places the prevalence of genuinely severe, refractory disease lower, around three to four percent. This group accounts for a disproportionate burden of exacerbations, hospitalizations, corticosteroid exposure, and healthcare costs [3]. Severe asthma, moreover, rarely occurs in isolation. In a substantial proportion of patients, it coexists with chronic rhinosinusitis with nasal polyps (CRSwNP). The two respiratory conditions powerfully illustrate the paradigm of the unified airway, representing a single integrated system in which the upper and lower respiratory tracts are deeply interconnected and share the same inflammatory process. The T2-driven process is governed by the common interleukin axis (IL-4, IL-5, and IL-13), elevated immunoglobulin E (IgE) levels, and tissue eosinophilia, underscoring the evidence that nasal and pulmonary conditions should be managed as a single, continuous disease spectrum [2,4]. This review focuses on severe lower airway disease, but treats concomitant upper-airway disease not as an incidental comorbidity but as an expression of the same inflammation, whose activity can modify and often constrain lung remission.

Against this background, the difficulty is not that remission is unattainable. Pooled analysis of biologic-treated severe asthma indicates that a meaningful minority of patients with that condition reaches clinical remission, about thirty to forty percent depending on the criteria applied [5]. The difficulty is that the term does not denote a stable quantity. In a systematic review, sixty-eight distinct definitions of clinical remission were identified, and the choice between a three- and a four-component definition alone shifted the pooled attainment estimate from thirty-eight to thirty percent [5]. When a single dataset can yield materially different remission rates depending on whether lung function is included, whether the corticosteroid-free interval is six or twelve months, and which symptom threshold is chosen, the headline figure of roughly one-third loses much of its meaning as a description of any individual patient or any individual drug. Consensus initiatives have proliferated in response, from the early expert framework of Menzies-Gow and colleagues [6] to national statements in Italy [7], the United States [8], and Japan [9], but so far they have multiplied definitions rather than converging on one. The upper airway has followed a similar trajectory, with the European Forum for Research and Education in Allergy and Airway Diseases (EUFOREA) and the European Position Paper on Rhinosinusitis and Nasal Polyps (EPOS) expert panel proposing an analogous definition of remission in CRSwNP [10].

Two consequences follow, and together they define the gap this review addresses. First, remission, as currently used, is not a single target but a family of targets with differing stringency, and treating them as interchangeable obscures rather than clarifies comparisons among biologics. Second, the headline attainment figures say little about the depth of the state they describe, conflating patients whose symptoms are merely controlled on treatment with the far smaller number in whom the underlying inflammation and its structural consequences have genuinely resolved. This review therefore does not aim to catalog remission rates drug by drug, an exercise that would inherit all the incomparability just described. Recent work has begun to move in this direction, most notably a framework that separates modifiable disease activity from fixed structural damage [11], and the account developed here complements it. It aims to reorganize the evidence around the proposition that asthma remission is a graded and multidimensional construct including clinical, biological, and functional axes, reached independently and often discordantly, whose measurable depth depends on where each biologic acts within the T2 cascade and is bounded by structural remodeling and by the distinction between suppressing and resetting the disease. We state from the outset that this framework is conceptual, has not been validated in clinical studies, and is offered to organize the evidence and generate hypotheses rather than as an established model. We use this framework to ask, for each biologic class, not simply how often remission is reported but how deep it plausibly runs, treating concomitant upper-airway disease as one factor that modifies that depth, and we calibrate throughout what the evidence establishes against what it can only suggest.

## 2. Materials and Methods

A narrative review was conducted in accordance with the Scale for the Assessment of Narrative Review Articles (SANRA) guidelines [12] to evaluate current evidence on remission as a treatment goal in adults with severe asthma treated with biologic therapies, with concomitant CRSwNP considered only as a comorbidity capable of modifying asthma remission rather than as a separate outcome. A comprehensive literature search was performed in the PubMed database through June 2026, using “severe asthma,” “remission,” and “biologic” as the primary search terms, complemented by supplementary searches pairing asthma remission with “chronic rhinosinusitis with nasal polyps,” with each individual biologic agent (omalizumab, mepolizumab, reslizumab, benralizumab, dupilumab, and tezepelumab), and with the concepts of remission definition, T2 biomarkers, and airway remodeling. We considered only English-language articles from peer-reviewed journals and, where available, prioritized systematic reviews, meta-analyses, and randomized controlled trials, complemented by observational and real-world studies reporting remission outcomes. Given the narrative nature of this review, we did not apply strict, predefined inclusion and exclusion criteria. This review was conceived as a narrative, conceptual synthesis and does not follow the PRISMA methodology, which is suited to a different question, that of estimating remission rates across studies, and which has recently been applied to that question in a systematic review and meta-analysis on which we draw throughout [5]. Instead, we selected articles for their relevance to the clinical, functional, biological, and complete remission of severe asthma and for the availability of essential information, such as sample size, the biologic evaluated, the remission definition applied, and remission rates. Data were synthesized qualitatively. Because reported remission rates proved to depend heavily on the definition applied, extracted findings were tabulated to record, for each study, the first author and year, study design, cohort size, the biologic evaluated, the specific remission definition and its depth and components, the resulting remission rate, and, where reported, the influence of concomitant CRSwNP.

## 3. What “Remission” Denotes, and Why the Field Cannot Agree

Any coherent account of remission must begin by distinguishing the concept from its measurement, because much of the confusion in the literature stems from conflating the two. As a concept, remission denotes the durable absence of meaningful disease activity, sustained over time rather than captured at a single visit. It is useful precisely because it sets a higher bar than control [2,6]. The two ideas diverge in what counts as activity and how it is evaluated. Clinical remission, the most frequently reported state, requires a stable absence of symptoms and exacerbations, along with freedom from systemic corticosteroids. Clinical remission can be achieved either while optimized maintenance treatment continues (inhaled therapies, long-acting muscarinic antagonists, and/or biologicals), so-called “remission on treatment,” or after treatment is withdrawn (“remission off treatment”) [6]. Remission on treatment is more accurately characterized as pharmacologically maintained disease suppression, rather than true disease inactivity or cure. Although not yet clearly and universally defined, remission on treatment is a uniform state that oversimplifies a complex clinical spectrum, involving clinical control maintained on optimized therapy (biologic + high-dose ICS/LABA/LAMA) or following successful de-escalation of background inhaled controllers while on biologics [2,13]. Unlike disease modification, which implies a permanent alteration in the natural history of the condition even after drug withdrawal, ongoing biological therapy actively suppresses persistent inflammatory pathways. Differentiating remission on treatment from remission off treatment is crucial. The former represents disease control dependent on continued pathway inhibition, whereas the latter reflects clearance or complete resolution of airway inflammation. Nearly all remission reported in this review is on treatment, meaning the criteria are met while the biologic and, in most cases, high-dose inhaled corticosteroids with a long-acting β2-agonist and sometimes a long-acting muscarinic antagonist are continued. Remission on treatment is itself heterogeneous with respect to the total burden of maintenance therapy, and we find it useful to distinguish four situations that current definitions do not separate: remission maintained on the biologic together with unchanged high-dose background therapy, remission maintained on the biologic after background inhaled therapy has been stepped down, which a controlled study has shown to be feasible for most patients receiving benralizumab [14], remission that persists on inhaled therapy alone after the biologic has been discontinued, and treatment-free remission. With the exception of the framework of Blaiss and colleagues, which explicitly allows remission to be recorded on or off treatment [8], published definitions require only the absence of oral corticosteroids and are silent about the dose of inhaled corticosteroids and the number of controllers, so that two patients meeting the same definition may carry very different treatment burdens. In this review, the term remission denotes remission on treatment unless stated otherwise, and we specify, wherever the source allows, the treatment on which each rate was observed.

Functional remission includes the restoration of organ function, operationalized in the lung as normal or stabilized spirometry [6], a proxy for airflow that says nothing about exercise capacity or the disability the disease imposes on daily life. Biological remission requires resolution of asthma-related inflammation, evidenced by normalization of biomarkers, including fractional exhaled nitric oxide (FeNO), blood or sputum eosinophils, and, in some definitions, IgE and periostin. Blood eosinophil count (BEC) serves as a critical index of systemic T2-high activity. Baseline elevations often predict a better response to anti-IL-5 or anti-IL-4R agents, while post-treatment suppression below 150 cells/μL, along with a decrease in sputum eosinophils to less than 2%, is a key threshold reflecting successful pathway antagonism and clinical asthma remission. Similarly, FeNO typically drops and stabilizes below 20 ppb during remission, serving as a valuable surrogate for tracking global pan-airway resolution and localized bronchospasm suppression. Total serum IgE levels, along with a longitudinal decline in allergen-specific IgE, reflect systemic atopic burden, indicating adequate target engagement during anti-IgE or anti-IL-4Rα protocols and a transition toward a controlled mucosal state [6,15]. However, biological remission is the hardest to achieve because subclinical airway hyperresponsiveness, obstruction, mast cell activation, or non-eosinophilic inflammation often persist after symptoms resolve [6,15]. These commonly monitored surrogate T2 biomarkers have notable limitations for assessing biological remission. Achieving complete resolution of airway inflammation requires tissue/gene-level confirmation to verify the absence of subepithelial remodeling, persistent mucosal edema, and residual neuroimmune or non-T2 pathways.

Complete remission, finally, encompasses all three domains, though it is not uniformly defined or validated in clinical trials [6]. Clinical, functional, and biological quiescence are distinct domains. A patient may occupy any one of them without the others, and clinical remission without biological remission and biological remission without clinical remission are both regularly observed. Only complete remission is nested by construction, because it requires all three. Reading these states as a complexity and a possible combination of different domains, rather than as competing definitions, is the first step toward making the literature comparable, because a study reporting clinical remission and a study reporting complete remission are not disagreeing about how often remission occurs; they are measuring different domains of the same phenomenon (Table 1). Since no consensus document has so far operationalized complete remission in a uniform way, we adopt throughout this review a single working definition, namely the concurrent fulfillment, for at least twelve months, of the clinical criteria (symptom control, absence of exacerbations and no oral corticosteroids), the functional criterion (normalized or stabilized lung function) and the biological criterion (normalization of T2 biomarkers), with or without a negative bronchial challenge. Where the studies discussed below use the word “complete” for a narrower construct, typically the four clinical and functional components without any biomarker, we say so and label it complete clinical remission. The whole framework, it should be stressed, is conceptual; it has not been validated in clinical studies, and it is offered as a way of organizing the evidence rather than as an established model.

This proposed multidimensional framework remains conceptual and has not been standardized. When Shackleford and colleagues examined biologic-treated severe asthma, they identified sixty-eight definitions of clinical remission and showed that the definitional choice was not a second-order detail but a primary determinant of the result, with attainment falling from thirty-eight percent under a three-component definition to thirty percent when lung function normalization was required [5]. Disagreement runs through every component. There is broad agreement that remission requires withdrawal of oral corticosteroids (OCS), but not on the minimum steroid-free interval, or on the symptom instrument and its threshold, with the Asthma Control Questionnaire (ACQ) applied at cut-points ranging from the conventional 1.5 to the far more exacting 0.75, a single choice that can substantially shift the apparent remission rate within one cohort [5]. The national and international consensus statements assembled over recent years, valuable as each is on its own terms, have so far encoded these differences rather than resolved them, so that the Italian [7], United States [8], Spanish [18] and Japanese [9] frameworks agree on the architecture of remission while differing on its thresholds (Table 2). The predictable result is that remission rates cannot be compared across studies without first reconstructing, and often failing to reconstruct, exactly which definition each applied.

This heterogeneity matters most where it is least visible, in the transition from clinical to biological remission. A patient may satisfy every clinical criterion, being free of symptoms, exacerbations, and corticosteroids and having preserved lung function, while residual eosinophilic inflammation persists in the airway wall [15]. The distinction is not academic, because it bears directly on the question clinicians and patients most want answered: whether treatment can eventually be spaced or withdrawn, a decision that turns on the depth of remission rather than its surface. Asthma remission is not always drug-induced, as it can occur spontaneously. A proportion of children with recurrent wheeze and early onset asthma experience spontaneous clinical remission, a reminder that the natural history of the disease is variable and that symptom-based remission can overlook persistent subclinical disease [15]. The natural history of childhood asthma includes a well-documented trajectory toward spontaneous remission, particularly during adolescence, whereas it is exceedingly rare in adult-onset severe asthma. Adult- and pediatric-onset asthma are two phenotypic presentations of asthma that differ markedly. Pediatric asthma is overwhelmingly driven by allergic, T2-high mechanisms and has a greater potential for reversibility, whereas adult disease often features persistent mucosal inflammation, fixed airflow obstruction, and complex non-allergic co-factors. Distinguishing therapeutic remission from spontaneous disease waning remains a key clinical challenge in pediatric cohorts.

The upper airway now traverses the same terrain the lower airway did a few years earlier. The EUFOREA and EPOS proposal defines remission in CRSwNP as a prolonged state of control, with at least twelve months without bothersome symptoms, without OCS or endoscopic sinus surgery, and without endoscopic signs of active disease [10], a structure that deliberately mirrors the logic used in asthma. The practical consequence for the present review is not that we measure sinonasal remission as an endpoint, but that active nasal polyposis in a patient nominally in asthma remission indicates persistent T2 activity in a contiguous compartment. Such a patient is in remission of the lung but not of the T2-mediated systemic disease. CRSwNP and asthma are a prototypical example of the united airway concept, in which upper- and lower-airway disease share a common pathophysiological substrate. CRSwNP is predominantly characterized by T2 inflammation that, rather than remaining confined to the sinonasal mucosa, closely interacts with the lower respiratory tract, contributing to the high co-occurrence of severe asthma. Recognizing CRSwNP as part of a unified airway disease has profound implications for disease remission. Monoclonal antibodies targeting key T2 drivers provide dual benefit, concurrently suppressing nasal polyp burden, restoring olfaction, and improving lower airway mechanics while reducing systemic exacerbation rates. Achieving true complete remission therefore requires evaluating outcomes across the entire respiratory tract, reinforcing the need for integrated upper- and lower-airway management strategies.

## 4. From Mechanism to Depth: What the Site of Action of a Biologic Can and Cannot Tell Us About Remission

What determines the remission any given biologic therapy can reach? Our proposed answer is partly geometric, and it needs to be stated with care. Where along the T2 inflammatory cascade a biologic intervenes determines, with reasonable confidence, how much of the downstream network is silenced and therefore which biomarkers shift under treatment [1]. Whether it also determines how deep a remission the agent can reach is a different and unproven proposition. The intuitive expectation that a more upstream target with broader inhibitory effects necessarily yields a deeper remission is a hypothesis generated by this framework rather than a conclusion supported by it. The clinical data reviewed in Section 6 do not support this expectation, since the highest clinical remission rates have been reported with agents acting downstream of the alarmins. What the site of action reliably predicts is the pharmacodynamic footprint of a drug, and this matters for remission because the biomarkers that define its biological grade are precisely the quantities that the footprint alters.

**Figure 1:**
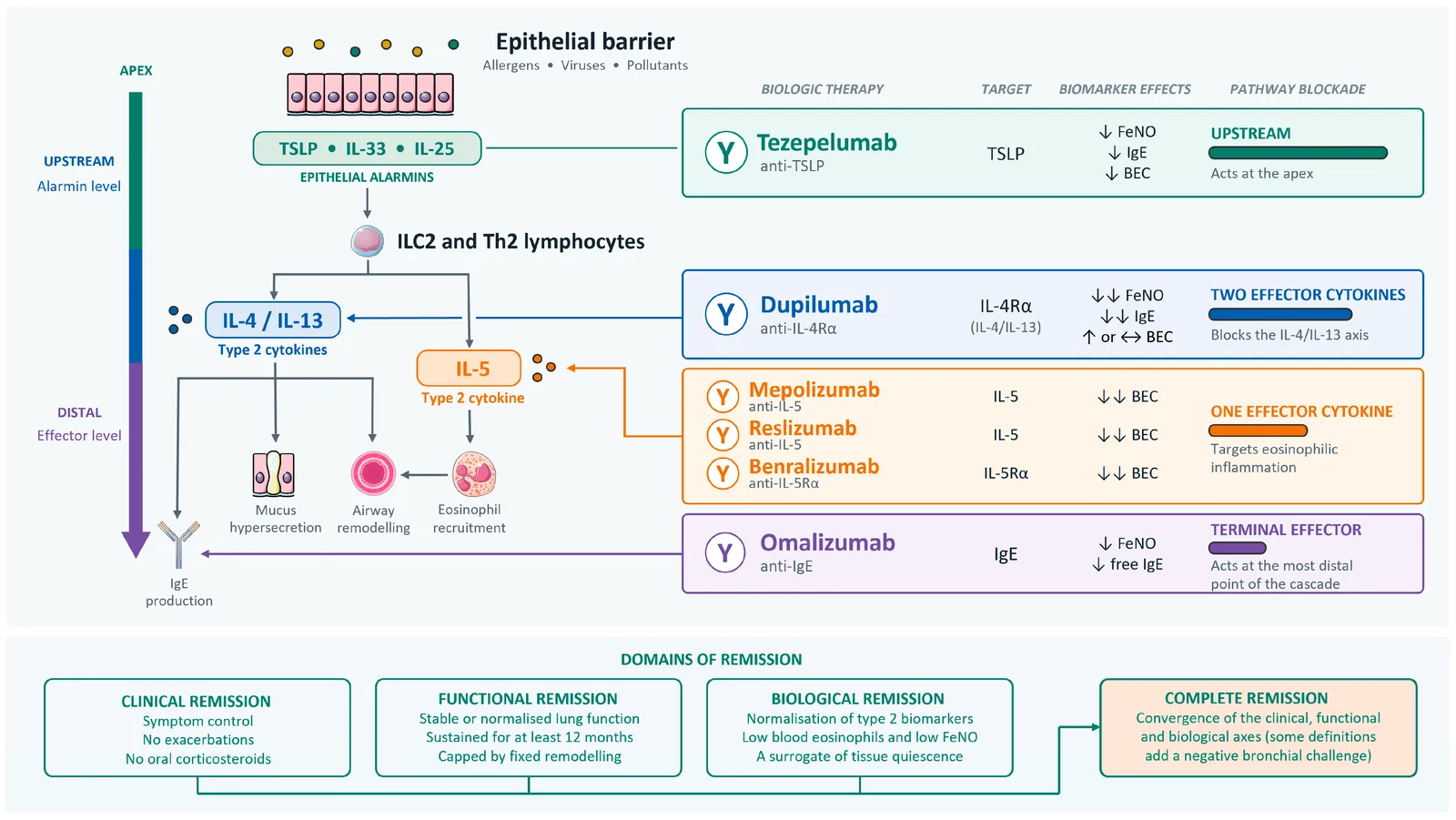
The type 2 (T2) inflammatory cascade in asthma and the level of action of biologic therapies. Epithelial injury by allergens, viruses, and pollutants releases the epithelial alarmins thymic stromal lymphopoietin (TSLP), interleukin (IL)-33, and IL-25, which activate group 2 innate lymphoid cells (ILC2) and T-helper 2 lymphocytes. These converge on two effector arms, IL-4 and IL-13, which drive immunoglobulin E (IgE) production and mucus hypersecretion, and IL-5, which drives eosinophil maturation, survival, and recruitment. Both arms converge on airway remodeling, to which eosinophils contribute by releasing pro-fibrotic mediators. The effector limb is depicted as two parallel and interacting axes rather than a single chain, and TSLP is one of three epithelial alarmins rather than a sole master switch. Each approved biologic is shown at the level of the cascade it intercepts, together with its molecular target, the direction of change in the biomarkers it moves during effective treatment, a single arrow denoting a modest and a double arrow a marked change, and the extent of pathway blockade it produces, from interruption at the alarmin level (anti-TSLP) to blockade of the terminal effector (anti-IgE). This ordering describes how much of the cascade each agent interrupts, and therefore which biomarkers move, rather than ranking the agents by clinical efficacy. Agents are listed by extent of blockade rather than by level in the cascade, since IL-4 and IL-13 lie at the same level as IL-5. Blood eosinophils may rise or remain unchanged under anti-IL-4Rα blockade, which inhibits their recruitment into tissue rather than their production; under anti-IgE, free rather than total IgE falls, since total serum IgE rises through accumulation of drug–IgE complexes. The lower panel shows the three domains of remission, clinical, functional, and biological, as parallel axes that can be reached independently and discordantly and that converge on complete remission, which requires all three. The domains are not consecutive steps; the panel describes dimensions of remission rather than the level at which each biologic acts, and the framework is conceptual and has not been clinically validated. BEC, blood eosinophil count; FeNO, fractional exhaled nitric oxide. Selected graphical elements were provided by Servier Medical Art (https://smart.servier.com (accessed on 10 August 2026)), licensed under CC BY 4.0 (https://creativecommons.org/licenses/by/4.0/).

The cascade itself is well characterized and can be summarized in functional terms. Epithelial injury from allergens, viruses, smoke or pollutants provokes the release of the epithelial alarmins thymic stromal lymphopoietin (TSLP), IL-33 and IL-25, which activate both dendritic cells and group 2 innate lymphoid cells (ILC2). Downstream of this alarmin signal, adaptive T-helper 2 lymphocytes and ILC2 converge on the same trio of effector cytokines, IL-4, IL-5 and IL-13, each with a distinct output. IL-4 and IL-13 drive B-cell class switching to IgE and, through the shared IL-4 receptor alpha subunit (IL-4Rα), promote goblet-cell metaplasia, mucus hypersecretion and the fibrotic signaling that underlies remodeling, while IL-5 governs the differentiation, survival and recruitment of eosinophils, which close the loop by releasing granule proteins and pro-fibrotic mediators that perpetuate epithelial damage [1]. The cascade is shown here as a linear sequence for clarity, but the effector limb is better described as two parallel, interacting axes rather than a single chain. The IL-4/IL-13 axis, which governs IgE synthesis, mucus, and remodeling, and the IL-5 axis, which governs eosinophils, are driven by the same upstream cells yet operate largely side by side and communicate at several points. IL-13 induces the eotaxins that recruit eosinophils into tissue; IL-4 sustains the T-helper 2 differentiation that produces IL-5; and eosinophils themselves release IL-13 and other mediators that feedback on the epithelium [1]. Nor is TSLP a single master switch. It is one of three epithelial alarmins, alongside IL-33 and IL-25, each able to activate ILC2 and sustain T2 inflammation without the others. TSLP also signals to dendritic cells, mast cells, and pathways that are not T2 at all [19]. Anti-TSLP therapy therefore acts at an upstream node with broad influence over the network, which is why it lowers FeNO, eosinophils, and IgE together. However, it does not regulate the entire T2 response from a single central point, and residual T2 activity under tezepelumab is measurable, as discussed in Section 5. What matters for remission is that this architecture is layered, with a small number of upstream alarmins feeding a broader downstream network, so that blocking the pathway at one level differs predictably in its biomarker signature from blocking it at another (Figure 1). This geometry describes how much of the network each blockade silences and therefore which biomarkers move under treatment, rather than ranking the agents by clinical efficacy.

The deepest and least settled question is whether any of these interventions resets the disease or merely holds it in check, because this distinction separates remission on treatment from remission after withdrawal. The evidence is sobering. In the COMET trial, patients with severe eosinophilic asthma who were controlled on mepolizumab for at least three years and then randomized to stop treatment experienced a prompt return of disease, with blood eosinophil counts rising roughly sixfold toward pre-treatment levels and a significantly shorter time to first exacerbation than those who continued [20]. This implies that anti-IL-5 blockade suppresses the eosinophilic program while it is administered, reflecting immunomodulation rather than an immune reset. On present evidence, therefore, remission with current biologics is overwhelmingly remission on treatment, a state of pharmacologically maintained suppression. The notion that prolonged therapy induces durable disease modification, though biologically plausible, remains a hypothesis rather than a demonstrated fact.

Structural remodeling imposes a ceiling on how far functional remission can extend. T2 cytokines drive airway smooth muscle proliferation, subepithelial fibrosis, goblet-cell metaplasia, and mucus plugging. These fixed changes set a floor for lung function that no degree of inflammatory suppression can lift once established. Biologics do more than switch off inflammation. Anti-IL-5 and anti-IL-5Rα blockade reduce airway smooth muscle mass and sub-basement membrane thickness. IL-4Rα blockade lowers mucus plugging and improves airway volume on functional imaging. Anti-TSLP reduces submucosal eosinophils and remodeling-associated matrix metalloproteinases, with several of these gains exceeding those achieved with corticosteroids [21]. The reversal is nonetheless partial, as a systematic review of biologic effects on occlusive mucus plugs found residual plugging after treatment in every study [22]. Functional remission is therefore attainable to the extent that the obstruction is inflammatory and reversible, and capped to the extent that it is structural and fixed. This is the strongest mechanistic argument for intervening early, before remodeling consolidates, and which is the same distinction between activity and damage that has recently been proposed as the basis for redefining remission altogether [11].

One implication of this multidimensional framework extends to the upper airway, because the cascade that governs the bronchial mucosa is the same one that produces nasal polyposis. When severe asthma coexists with CRSwNP, the polypoid sinus serves as a reservoir of ongoing T2 activity in which barrier disruption and tissue remodeling may persist even as the same agent efficiently silences bronchial inflammation [1,23]. The link between the two compartments is not only anatomical. The united-airway concept rests on the observation that the nasal and bronchial mucosa share the same epithelial lining, the same barrier vulnerability to allergens, microbes and pollutants, and the same effector program of IL-4, IL-5 and IL-13, IgE synthesis and tissue eosinophilia, so that a T2 process established in one compartment reflects and reinforces the process in the other [4,23]. The connection is mediated in part systemically. Experimental allergen challenge confined to the nose induces eosinophilic inflammation and adhesion-molecule expression in the bronchial mucosa of patients who were not challenged there, along with a rise in circulating eosinophils, indicating that upper-airway inflammation feeds a systemic T2 signal rather than remaining a local event [24]. The same circulating pool of eosinophils supplies both compartments, and blood eosinophils and FeNO, the markers used to define biological remission, read this shared systemic activity rather than either compartment alone. This is why the severity of nasal polyposis tracks the severity and eosinophilic character of the accompanying asthma, why polyps mark a T2-high, biologic-responsive endotype, and why a single agent acting on the shared pathway can bring both compartments toward quiescence, as reviewed in Section 6 [4]. The remission attained in the lung need not, therefore, be matched in the nose, and unresolved upper-airway disease is best read as a marker that the underlying process has not been brought fully to rest, a factor that both signals and limits the depth of asthma remission rather than an independent target.

## 5. Biomarkers and the Limits of “Biological” Remission

Because biological remission is defined by biomarker normalization, the concept’s credibility depends on whether available biomarkers truly reflect quiescence of the underlying disease. Three markers carry most of the weight in current practice: the blood eosinophil count, FeNO, and total or allergen-specific IgE, each noninvasive, reproducible, and a validated predictor of response. It is unsurprising that definitions of biological remission typically require a blood eosinophil count below 150 cells per microliter together with FeNO below 20 to 25 parts per billion (ppb) [25]. The difficulty, as the multidimensional framework makes clear, is that these three biomarkers do not capture the same asthma phenotype, since each reflects on a different arm of the inflammatory cascade. For biologic therapies, each marker is a direct pharmacodynamic readout of the drug’s target rather than an independent witness to the state of the airway. Eosinophil depletion with an anti-IL-5 receptor antibody, the fall in FeNO with IL-4 receptor blockade, and the fall in free IgE with omalizumab are the effects these drugs produce, and they occur largely irrespective of clinical response. Thus, normalization of a targeted biomarker demonstrates target engagement rather than resolution of the inflammatory process. Biological remission in the strict sense, meaning quiescence of the underlying disease, would require evidence independent of the drug’s direct footprint, such as normalization of markers the drug does not directly target, tissue-level or imaging evidence of resolved inflammation, or persistence of quiescence after withdrawal. None of the biomarkers in routine use meet this standard, and we therefore use the term biomarker normalization for what current definitions actually measure and reserve biological remission for the state they are meant to approximate.

This asymmetry is measured, not merely theoretical. Under tezepelumab, the most upstream agent, clinical remission at one year was achieved by 36% of a real-world cohort and by 55% of its T2-high patients, while biological remission was attained by 38% and both states together by just 15%, with FeNO and eosinophils falling substantially but incompletely [26]. The gap between clinical and biological remission is therefore real even when the drug acts at the apex. Reported differences in biological remission among biologics thus reflect, in large part, the arithmetic of which pathway each drug silences rather than any real difference in the depth of control.

The most instructive evidence complicates the assumption that biomarker-defined remission is simply a deeper, better state. In the largest analysis addressing the question, drawn from the United Kingdom Severe Asthma Registry, 19% of patients reached biological remission defined by suppressed FeNO and eosinophils. However, this group derived no additional benefit over the remainder in exacerbation reduction, corticosteroid exposure, lung function, or symptom control. More revealing, the patients who achieved it were those who had been less T2-high before treatment began, with long disease duration, macrolide use, and a smoking history predicting attainment, while higher baseline T2 biomarkers predicted its absence [27]. The uncomfortable implication is that crossing the biomarker threshold can identify patients whose disease was never strongly eosinophilic, rather than patients in whom a biologic has driven florid inflammation into quiescence, so that biological remission, as currently operationalized, is not a straightforward index of depth. The same analysis found that suppression of T2 biology remained important for gains in lung function, which means a low eosinophil count and a low FeNO reading are necessary conditions for confidence in remission rather than sufficient definitions of it [27].

These systemic surrogates cannot capture what the depth framework identifies as the harder targets: tissue-level quiescence, restoration of the epithelial barrier, and reversal of structural remodeling. The available bronchoscopic evidence for the most upstream biological agent illustrates this gap. In the CASCADE trial, tezepelumab reduced airway submucosal eosinophils and airway hyperresponsiveness but did not significantly change submucosal neutrophils, T cells, or mast cells, so that a normal blood or breath biomarker can coexist with a cellular infiltrate that has resolved only in part [28]. The biomarker most directly tied to this structural dimension is serum periostin, an extracellular matrix protein induced by IL-4 and IL-13 that correlates with airway remodeling and fixed airflow limitation and might, in principle, mark the resolution of structural disease [29]. Its translation into practice has stalled because of poor accuracy relative to blood eosinophils, wide variability, and the absence of a standardized assay. The wider field of emerging markers, from sputum inflammatory profiles to transcriptomic and volatile-organic-compound signatures, remains promising for endotyping but is unvalidated for the specific task of confirming remission [25]. It is worth stating plainly, against a tendency to present these assays as near-ready, that none has so far been shown to define or predict remission with the reliability a treat-to-target strategy would require.

A final limitation follows from the systemic nature of these readouts. FeNO and blood eosinophils reflect the circulating and lower-airway compartments, and a patient may meet both thresholds even as T2 activity persists at the tissue level, including in a coexisting polypoid sinus that no routine laboratory value interrogates [25].

Taken together, these considerations answer a question the framework raises directly: whether blood eosinophils, FeNO, and IgE are sufficient to indicate complete resolution of airway inflammation. They are not. They are necessary, inexpensive, and reproducible sentinels of the two main T2 axes, but each is a systemic or exhaled surrogate of a tissue process; each is directly modulated by at least one biologic; none reports on mast cells, the epithelial barrier, or the sinus compartment; and none has been validated against a histological or imaging standard of quiescence. Biological remission defined by these three markers is therefore a working approximation, and reported biological remission must be interpreted in light of which biomarkers were available to define it.

## 6. The Clinical Evidence, Read in Depth

Evidence on remission with biologic therapy has accumulated almost entirely in adults and over the past few years, and it should be read with the two caveats already established. First, any single remission rate reflects both the drug and the definition, so figures are comparable within a study but rarely across studies [5]. The second is that the depth of remission, not merely its frequency, may distinguish the agents, and the biological dimensions of remission plausibly track where in the T2 cascade each one acts [1]. We therefore examine each class to ask how deep the remission it induces plausibly reaches, how durable it is, and how far concomitant upper-airway disease qualifies it, drawing together the studies summarized in Table 3.

### 6.1. Anti-IgE Blockade

Omalizumab occupies the most distal position among the approved biologics, neutralizing IgE downstream of the effector cytokines. In a real-world cohort followed for five years, clinical remission rose steadily rather than plateauing, from 25.9% during the first two years to 34% in years three and four and 47.4% by the fifth year [30]. A remission that accrues slowly over years is more consistent with the gradual resolution of structural and cellular disease [21] than with the rapid switching-off of an inflammatory signal. What makes the same study telling is a negative result, namely that no baseline clinical or demographic characteristic predicted who would reach remission [30]. This unpredictability is difficult to reconcile with the assumption that remission can be forecast from baseline T2 markers, and it supports the view that a drug acting too far downstream to be captured by upstream measurements may not be predictable from baseline. It should be read with restraint, since the cohort was small, and the broader registry evidence tempers the picture, finding that higher baseline lung function and asthma control, lower body mass index, and the absence of obesity, anxiety, or depression are associated with clinical remission across biologics in registry cohorts treated with mepolizumab or omalizumab [40].

The depth of remission is nonetheless limited by the inadequacy of available biomarkers for the various asthma phenotypes. Because omalizumab does not deplete eosinophils or affect their count, biological remission cannot be fully assessed or compared with other biologics [1]. This is not a failure of the drug, but a direct consequence of its selective mechanism of action, and it exemplifies the central argument that clinical and biological remission are distinct domains rather than competing measures of the same thing. For omalizumab, symptomatic and functional remission is attainable and durable, while resolution of underlying allergic inflammation remains largely unexplorable, a limitation that matters most in patients whose disease, including any coexisting nasal polyposis, is also driven by eosinophils.

### 6.2. Anti-IL-5 and Anti-IL-5Rα Blockade

Mepolizumab and reslizumab neutralize circulating IL-5, while benralizumab binds its receptor and directly depletes eosinophils [1]. This mechanism of action leaves the IL-4 and IL-13 arm intact. Clinical data support the first half. Across real-world cohorts, one-year clinical remission occurs in a quarter and two-fifths of patients, with benralizumab reaching 35.8% and mepolizumab 23.6% in one multi-biologic series [41], and mepolizumab and benralizumab reaching 30% and 40%, respectively, for a four-component definition that its authors termed complete remission, without any biomarker criterion [31]. These figures come from cohorts assembled for different indications and under different criteria and should not be read as a head-to-head comparison of the two agents. More informative than any single figure is a signal that recurs across the class, namely that remission rates rise with continued treatment, from 52% at one year to 73% at three with mepolizumab under a definition requiring lung-function stabilization [32], and from 86.9% at one year to 91.3% at four with benralizumab, with the proportion meeting the stricter criteria of complete clinical remission, in the SANI sense of all four clinical and functional components, rising from 65% to 71.4% over the same period [33]. A remission that deepens over years recurs too consistently across agents to be dismissed as a cohort artifact.

The same evidence shows how much the reported rate depends on the definition. In the pooled post hoc analysis of the pivotal benralizumab trials, the same patients yielded a three-component remission rate of 39.2% and a four-component rate, adding lung function, of 33.4% [34]. One drug, one dataset, and a spread of several percentage points depending only on how remission was operationalized: this is the heterogeneity argument demonstrated within a single trial program.

This class appears to excel in biological remission. Because these agents drive eosinophils to zero, they inevitably score highest on any definition of biological remission centered on eosinophil suppression. However, that apparent advantage is partly an artifact of measurement, and biomarker-defined remission does not, by itself, confer better clinical outcomes and may flag patients whose disease was less floridly T2 to begin with [27]. The honest reading is that these agents achieve profound and measurable suppression of the eosinophilic program, which represents real depth along one axis, without necessarily amounting to global disease quiescence.

It is in this class that concomitant upper-airway disease shows its clearest effect, and the direction is instructive. Far from limiting remission, comorbid CRSwNP predicts a better multidomain response. In real-world analysis, CRSwNP is an independent predictor of clinical remission with both benralizumab and mepolizumab [41]. In the ANANKE program, patients with comorbid nasal polyps achieved greater exacerbation reduction, a faster lung-function response, and a higher probability of sustained remission, with CRSwNP emerging as a significant predictor of remission itself [35]. This is not paradoxical, since nasal polyposis marks a more purely eosinophilic, T2-high endotype, precisely the disease that eosinophil depletion resolves most completely. Thus, the upper-airway comorbidity functions here as a positive marker of the endotype these drugs were built for.

None of this evidence supports the durability off-treatment. When long-term mepolizumab was stopped in the controlled setting of the COMET trial, eosinophil counts rebounded roughly sixfold toward baseline, and exacerbations resumed within weeks [30]. The remission delivered by this class, however deep along the eosinophilic axis and however favorably modified by a T2-high upper airway, remains a state maintained by continued dosing rather than a cure.

### 6.3. Anti-IL-4Rα Blockade

Dupilumab blocks the IL-4Rα subunit, thereby inhibiting signaling through both IL-4 and IL-13 and targeting a broader segment of the T2 inflammatory cascade [1]. Its distinguishing feature is that it produces the widest divergence between clinical and biological remission of any biologic in use. Clinically, performance is strong and durable. In the pooled post hoc analysis of the QUEST and TRAVERSE programs, the largest controlled dataset available for this agent, a substantial group of dupilumab-treated patients reached on-treatment clinical remission, which was sustained through the open-label extension [36]. Smaller real-world series point the same way, with three-component clinical remission of 68% and four-component remission of 57% at twenty-four months in one post hoc analysis [37], and asthma remission rising from 85.7% at twelve months to 100% at twenty-four in a cohort assembled around comorbid nasal polyposis [38]. This last figure should not be read as a remission rate for severe asthma, since fewer than a third of that cohort had severe disease and the upper airway was the dominant indication for treatment.

The picture changes abruptly when the definition is deepened. In the same 24-month cohort, complete remission, defined by the additional resolution of airway inflammation and a negative airway hyperresponsiveness test, was achieved by only 7%, an eightfold decline from the four-component rate within a single dataset [37]. It would be a mistake to read this collapse as evidence of a shallow remission. Dupilumab does not deplete circulating eosinophils and may transiently raise them, since it blocks their recruitment into tissue rather than their production; so a definition of complete or biological remission anchored to a low eosinophil count fails almost by construction, even in patients whose disease is clinically and functionally quiet [1]. On the axis it actually targets, dupilumab drives down FeNO and IgE and acts on the structural dimension that eosinophil counts cannot see, reducing mucus plugging and improving airway volume on functional imaging [21]. What looks like a failure of biological remission is, in fact, deep suppression of the IL-4 and IL-13 arm, mismeasured by a biomarker belonging to a different asthma endotype.

This is where the argument of the review comes together. Eosinophil-directed agents show high biological remission rates because the standard definition rewards eosinophil depletion, whereas dupilumab posts low rates because the same definition penalizes its mechanism. Yet, both observations reflect measurement rather than the true depth of control. A remission construct that cannot rank these two classes without contradicting itself is not fit to serve as a treatment target.

Concomitant upper-airway disease improves dupilumab outcomes more favorably, and more visibly, than any other agent. A higher burden of comorbid nasal polyposis independently predicted four-component asthma remission in the cohort above [37]. When both compartments were followed together, they converged under a single agent: asthma remission reached 100%, while sinonasal remission climbed from 57% at twelve months to 88% at twenty-four, in a small cohort whose asthma was predominantly moderate [38]. Treating the upper airway as a modifier rather than an endpoint, the coexisting polypoid sinus is not a hidden reservoir that quietly caps asthma remission but a compartment brought toward quiescence on a parallel trajectory, which is the united-airway logic in its clearest current illustration rather than proof of its general validity. The familiar ceiling nonetheless applies, since nothing in the present data indicates that IL-4Rα blockade resets the disease rather than suppressing it while administered.

### 6.4. Anti-TSLP Blockade

Tezepelumab neutralizes TSLP, one of the three epithelial alarmins at the apex of the cascade. On mechanistic grounds, it is the broadest of the approved agents, although, as argued above, it does not block the entire T2 response, and it is the only one with activity in both T2-high and, to a lesser extent, T2-low disease [1]. Its broad effect suggests it should lower FeNO, blood eosinophils, and total IgE alike [1], and thus more readily achieve biomarker-defined biological remission across multiple axes than agents acting on a single arm. Real-world data confirm that FeNO and eosinophils decrease with treatment [22]. Breadth of coverage, however, describes which markers change, not a claim of greater clinical benefit.

What tezepelumab does not do is close the gap between clinical and biological remission. In the same cohort, 36% reached clinical remission overall, and 55% of T2-high patients did, while 38% reached biological remission and only 15% reached both [26]. The persistence of this divergence at the apex of the cascade is instructive, because it shows that the shortfall of biological remission beneath clinical remission cannot be dismissed as an artifact of agents that block only one arm. Even the drug that suppresses the pathway before it branches leaves residual T2 activity in a substantial fraction of clinically quiescent patients, so that incomplete resolution is intrinsic to on-treatment remission with current therapy. These figures also expose the limits of any strictly nested reading of the grades, since biological remission was slightly more frequent than clinical remission and the two overlapped in fewer than half of those who reached either, reflecting two independent axes rather than consecutive levels. This is one reason we describe the framework as multidimensional.

The remaining features reinforce the recurring patterns. Remission is concentrated in the T2-high endotype, reaching 55% there versus 19% in T2-low disease [26], and rates rise modestly over time, from 28.5% in the first year to 33.5% in the second in the pooled analysis of the pivotal trials [39]. That controlled analysis provides necessary calibration, since on-treatment remission with tezepelumab exceeded the placebo only modestly and did not reach statistical significance, with more than a fifth of placebo recipients also meeting the criteria on optimized background therapy [39]. That more than a fifth of placebo-treated patients with severe, uncontrolled asthma met a remission definition within a year deserves comment, and we read it in three ways. First, enrolment in a trial optimizes background treatment itself, with protocol-mandated medium- to high-dose inhaled corticosteroids, monitored adherence and inhaler technique, and regular review, so that a proportion of patients whose asthma was uncontrolled largely because of treatment delivery regain control on optimized conventional therapy alone. Second, entry required recent exacerbations, and regression toward the mean ensures that some of the worst years are followed by quieter ones irrespective of treatment. Third, the definition applied in that analysis used the less exacting ACQ-6 threshold of 1.5 and required only stable rather than normalized lung function, both of which raise the attainable rate. The dupilumab program shows almost the same placebo figure, 22.2% [36], suggesting this reflects trial populations and definitions rather than either drug. The implication for the framework is that remission on treatment has to be interpreted against the background rate achievable with optimized conventional therapy, that the incremental contribution of a biologic is smaller than the absolute rate implies, and that uncontrolled real-world cohorts, in which starting a biologic usually coincides with a general optimization of care, will tend to overstate what the antibody itself contributes. Complete remission remains rare, of the order of one in eleven in a prospective study [17], in which only a quarter of the patients in clinical remission also met the biomarker criteria, and although tezepelumab is unusual in having been examined after treatment cessation, the available data give no indication that upstream blockade resets the disease rather than holding it in suspension [45].

Across the four classes, clinical remission is attainable for roughly a quarter to a half of treated patients in the first year and tends to deepen thereafter; complete and biological remission remain the exception, and no agent has demonstrated durable remission once treatment stops. The apparent differences between drugs dissolve substantially when the definition’s stringency and the biomarker axis it privileges are held constant, leaving a residuum of genuine mechanistic difference best described in terms of depth and reach. Notably, the reported rates do not follow a cascade order, since the highest clinical remission figures come from agents acting downstream of the alarmins rather than at the apex, and the only controlled estimate available for the most upstream agent exceeded placebo without reaching significance [39]. That the rates do not rank in cascade order is itself evidence against the simple proposition that a more upstream target yields deeper remission, and it is one reason we present the relation between site of action and depth as a hypothesis rather than a finding. The classes differ most reliably in how concomitant upper-airway disease is accounted for: as a liability that systemic markers may mask for the distal anti-IgE agent, as a favorable marker of the responsive endotype for the eosinophil-directed agents, and as a co-treated compartment for anti-IL-4Rα blockade.

## 7. The Registry-Level Picture: Convergence and Its Lesson

Class-by-class evidence gains credibility when large registries tell the same story, and on the whole they do. The most complete synthesis found clinical remission in 38% of biologic-treated patients under a three-component definition and 30% once lung function was added, framing the realistic expectation as roughly one-third [5]. Real-world registries align with this estimate. In the United States CHRONICLE cohort, the point prevalence of on-treatment clinical remission rose from 22.3% between months twelve and thirteen to 34.3% by months forty-seven to forty-eight, with a median time to remission of thirty months [43]. In the United Kingdom Severe Asthma Registry, 18.3% met the primary clinical remission definition at one year, with rates ranging from 12% to 21% depending on the definition used [42]. A nationwide Korean cohort reported clinical remission in 18.3% at twelve months but complete clinical remission, adding lung function, in only 5.9% [16]. The largest international analysis, drawn from twenty-three countries in the International Severe Asthma Registry, reproduced the same gradient within a single dataset, with 33.5% meeting a three-domain definition and 20.3% a four-domain definition in the year after starting a biologic [44]. The consistency across continents, of a clinical remission rate near a fifth to a third that deepens with time, and of a rate several-fold lower once lung function and, where measured, biomarkers are included, is reassuring evidence that the phenomenon is real.

The registries also convert the review’s central argument from assertion to measurement. In the pooled synthesis, sixty-eight distinct definitions of clinical remission were identified, and the effect was quantified directly. In a single cohort assessed against twelve four-component definitions, the remission rate ranged from 20.5% to 41.0%, and in a single mepolizumab cohort assessed against four three-component definitions, from 18.9% to 34.9% [5]. When a single population, treated with a single drug, can be reported as achieving remission in either one-fifth or two-fifths of patients, depending only on the definition chosen, the definition is the dominant source of variance in the literature, and its harmonization is the precondition for remission to function as a treatment target.

Two additional signals recur and point in the same direction. The predictors of remission are strikingly consistent, namely a T2-high biomarker profile, shorter disease duration, lower corticosteroid burden, and the absence of obesity. The United Kingdom registry found sevenfold higher adjusted odds of remission in T2-high patients and a roughly one-seventh reduction in the likelihood of remission for each additional decade of disease [42]. This gradient was reproduced almost exactly in the international registry, where the odds of four-domain remission fell by about one-seventh for each additional decade [44]. The same time-dependence seen drug by drug appears again, with remission accruing over years rather than at once. Together, these findings make a coherent case that asthma remission is limited by how far the disease has progressed when treatment begins, and that intervening earlier is the most plausible lever for deepening remission. The registries also measure clinical remission almost exclusively because systemic biomarkers are rarely available at this scale, so biological remission remains essentially unmeasured at the population level.

## 8. Unmet Needs and Future Directions

The unmet needs that follow from this analysis are, for the most part, not therapeutic. Existing biologics already deliver clinical remission to a substantial minority and deepen it over time. The first and most consequential need is definitional. A remission construct that varies by twenty percentage points within a single cohort cannot support a treat-to-target strategy [5]. What is required is a single, internationally agreed definition applicable to each biologic, explicitly stratified by domains, from clinical through functional and biological remission, with harmonized thresholds, a common minimum duration, and ideally one that also separates what remains modifiable from what has already become fixed [11]. The proliferation of national consensus statements, valuable individually, has so far multiplied definitions rather than converging them [7,8,9], and convergence toward a globally standardized definition is now the priority [2]. Such a definition should also be explicit about the treatment on which remission is recorded, ideally grading the burden of maintenance therapy from unchanged high-dose background treatment through stepped-down inhaled therapy to biologic-free and treatment-free states, because a remission that is silent about what sustains it cannot tell a clinician when de-escalation is safe.

The second need is for biomarkers adequate to the deeper endpoints. The systemic markers currently used to define biological remission are endotype-specific, so a single eosinophil-based definition rewards one drug class and penalizes another, and none captures the tissue-level and structural quiescence that separates deep from superficial remission [25]. A usable definition of biological remission will have to be axis-aware, distinguish biomarker normalization from the tissue-level quiescence it is meant to approximate, and the field needs validated markers of remodeling reversal [21] and of durable immune quiescence, together with instruments able to assess the upper airway non-invasively when nasal polyposis coexists, before biological remission can be measured at the scale at which it would be applied.

The third need is to resolve the question of durability, which the present evidence leaves open in the most consequential way. Every class examined shows remission maintained on treatment, and the one controlled withdrawal study available showed prompt relapse and a sixfold return of eosinophils when treatment stopped, the signature of suppression rather than of a reset [20]. Whether earlier or longer intervention can convert suppression into genuine disease modification, and thus permit safe withdrawal, remains unknown, and it is the question most worth answering. The consistent registry finding that shorter disease duration predicts remission makes the window-of-opportunity hypothesis plausible enough to warrant dedicated prospective trials of early intervention with remission, and eventual treatment-free remission, as the pre-specified endpoint [42]. Alongside this sit two narrower gaps, namely the near-absence of remission data in children, in whom even the definition remains unsettled [46], and the lack of options for T2-low disease, which only Tezepelumab reaches and only weakly [26].

The pediatric gap deserves its own discussion, because the framework developed here was built on adult evidence and cannot be transposed to children unchanged. Three differences intervene. First, the lung continues to grow throughout childhood and adolescence, and persistent childhood asthma is associated with reduced growth and early decline in lung function in a large proportion of patients [47]. Therefore, what counts as normalized or stabilized lung function, and thus as functional remission, is a moving target that must be interpreted against expected growth rather than a fixed predicted value. Second, spontaneous remission is common. A large proportion of children with asthma become symptom-free during adolescence without any biologic intervention. However, lung function and bronchospasm often remain abnormal in those who appear to have remitted, meaning that in children the natural history itself produces clinical remission at a rate that would be attributed to treatment in an adult cohort, and that symptom-based remission is an even less reliable proxy for disease quiescence than it is in adults [15,48]. Third, the phenotypic mix is different, with a predominance of early onset, allergen-driven, IgE-mediated disease, whereas late-onset eosinophilic asthma, the phenotype in which nasal polyposis and eosinophil-directed remission are most prominent, is largely an adult condition [48]. Therefore, the biomarker axes on which biological remission is defined, and their relation to the agents that move them, do not carry the same meaning. For these reasons, and because pediatric remission lacks even a consensus definition [46], the depth-stratified reading proposed here should be regarded primarily as an adult framework whose extension to children will require pediatric definitions anchored to lung growth, to the probability of spontaneous remission, and to the phenotypes that predominate at that age.

A fourth need is distinct because every definition surveyed here is built from symptoms, exacerbations, corticosteroid use, and spirometry, and none measures quality of life or what patients themselves recognize as recovery. Exercise capacity, physical activity, quality of life, and disability lie outside the construct, even though adults with severe asthma are consistently less active and more limited than their healthy peers, and such limitation may persist even when the disease is pharmacologically quiescent. Adults with severe asthma are less physically active and have a lower exercise capacity than healthy peers, and both are associated with airflow limitation, symptom burden, and comorbidity [49], while a recent real-world analysis found that daily step counts remained roughly two-fifths lower than in healthy controls in patients who had been on biologic therapy for at least two years, despite the improvements in symptom scores and quality of life that these drugs reliably produce [50]. Pulmonary rehabilitation targets this residual burden, improving exercise capacity and health-related quality of life in severe and uncontrolled disease [51], and real-world data show that those referred carry the heaviest symptom load and the greatest functional impairment [52]. Whether structured exercise training can deepen remission itself, or only make its absence more tolerable, has never been tested [53]. A depth-stratified definition that ignores functional capacity risks certifying as remission a state in which the patient remains substantially disabled. Beyond traditional clinical and biochemical markers, a comprehensive evaluation of treatment efficacy requires a deeper integration of patient-centered outcomes. From a holistic clinical perspective, disease remission should not merely reflect control of symptoms, biomarker levels, or organ-specific pathology, but should also translate into meaningful improvements in daily functioning and well-being. Incorporating standardized measures of exercise capacity (such as the 6-min walk test or cardiopulmonary exercise testing) and validated patient-reported outcome measures for disability and health-related quality of life provides a more accurate picture of asthma remission. Recognizing these domains as key components of true disease remission allows clinicians to optimize therapeutic strategies, address residual patient burden, and align clinical benchmarks with outcomes that matter most to patients.

This review has limitations that affect how its conclusions should be read. It is a narrative rather than a systematic review, follows SANRA rather than PRISMA, does not pool data, and is therefore subject to selection bias in the studies it emphasizes. Much of the remission evidence is observational and uncontrolled, drawn from cohorts of varying size and definition. The very heterogeneity that is the review’s subject also constrains its synthesis, since figures assembled under different definitions cannot be meaningfully combined. Reports of remission are, moreover, more likely to be published when positive. These constraints temper the precision of any numerical claim made here, but they do not weaken the central argument, which concerns the structure of the remission concept rather than the exact value of any rate. If anything, the limitations themselves reinforce the difficulty of comparison. Its central proposal, that remission be understood as a multidimensional, depth-stratified construct whose measurable biological grade depends on where a biologic acts, is a conceptual framework that has not been validated prospectively. Several of its mechanistic interpretations, above all the relationship between site of action and depth of remission, are hypotheses that the available evidence can motivate but not confirm. A PRISMA-compliant systematic review with a registered protocol is the appropriate instrument for quantifying remission rates across definitions and agents and would complement rather than replace the conceptual work attempted here. Our group is currently undertaking such a review as a separate, complementary project, with a protocol submitted for registration in PROSPERO, to estimate remission rates by definition, by biologic class, and over time.

## 9. Conclusions

Biologic therapy has made remission a legitimate goal in severe asthma, achievable in clinical terms for roughly a third of treated patients and deepening with continued treatment [5]. This is a genuine advance over an era when symptom control was the ceiling of ambition. This review argues that the principal obstacle now standing between remission and its use as a treatment target is conceptual rather than pharmacological. Remission is a multidimensional state in which clinical, functional, and biological quiescence are distinct axes that can be reached separately and discordantly, and in which complete remission, understood as the convergence of all three, has yet to be uniformly defined or validated in clinical studies. Where a biologic acts in the T2 cascade determines which biomarkers change with treatment [1], and this, rather than any demonstrated relation between site of action and depth of remission, is what the evidence establishes. Two limits that the headline rates obscure apply to every class: the persistence of structural remodeling [21] and the fact that current agents suppress the disease rather than resetting it [20], so that remission with current biologics is a pharmacologically maintained state and not evidence of disease modification. Read through this lens, the apparent disagreements between drug classes largely dissolve into differences in definition and biomarker axis, and the low rates of complete and durable remission emerge as the honest and consistent signal. Of these conclusions, the heterogeneity of definitions, the dependence of reported rates on the definition applied, the prompt relapse after withdrawal, and the gap between clinical and biomarker-defined remission rest on clinical evidence, whereas the depth-stratified framework itself, and the proposition that an upstream target permits a deeper remission, are interpretations offered to organize that evidence and to generate hypotheses.

The priority, therefore, is a harmonized, depth-resolved and axis-aware definition of remission, explicit about the treatment burden on which remission is recorded and inclusive of the patient-centered dimensions of recovery that current criteria omit, against which any therapy can be judged on comparable terms [2], coupled with biomarkers capable of confirming the deeper endpoints [25] and with trials designed to test whether earlier intervention can turn suppression into durable, treatment-free remission [42]. Concomitant upper-airway disease should be incorporated into this framework as a factor that influences remission, favorable when it marks a responsive T2-high endotype and limiting when it signals residual activity that systemic markers miss. Until these steps are taken, the field will continue to report a reassuring headline figure that means different things in different hands, and remission will remain a term applied with more confidence than the underlying measurements justify.

## Figures and Tables

**Table 1:** The four domains of asthma remission. The clinical, functional, and biological domains are distinct axes that can be reached independently and discordantly rather than consecutive steps, and only complete remission is nested by construction, since it requires that the three sets of criteria be met together. The framework is conceptual and has not been clinically validated. Thresholds are those most commonly applied in the literature but are not universally agreed.

Grade	Domain of Quiescence Assessed	Typical Operational Criteria	Biological Axis	Attainability
Clinical	Stable absence of symptoms and exacerbations; OCS withdrawal	ACT ≥ 20 and/or ACQ < 0.75–1.5; no exacerbations; no OCS; ≥12 months	None (clinical)	Frequent: ~1/5–1/3 at 1 year, rising over time
Functional	Restoration/stabilization of organ function	FEV1 ≥ 80% predicted or stable vs baseline	None (functional)	Lower than clinical alone (capped by fixed remodelling), e.g., 5.9% vs. 18.3% when lung function is added [16]
Biological	Quiescence of the underlying T2 inflammation	FeNO < 20–25 ppb; BEC < 150 (or <300) cells/µL	Agent-dependent: eosinophils (anti-IL-5/5R), FeNO/IgE (anti-IL-4R), both (anti-TSLP)	Does not coincide with clinical; axis-dependent
Complete	Sum of clinical, functional and biological dimensions	All the above, sometimes with negative bronchial hyperresponsiveness	Multi-axis	Rare: e.g., 9.3% [17] and 7% under the strictest criteria

ACQ, Asthma Control Questionnaire; ACT, Asthma Control Test; BEC, blood eosinophil count; FeNO, fractional exhaled nitric oxide; FEV1, forced expiratory volume in 1 s; IgE, immunoglobulin E; IL, interleukin; OCS, oral corticosteroids; ppb, parts per billion; T2, type 2; TSLP, thymic stromal lymphopoietin.

**Table 2:** Definitions of clinical remission of asthma across major consensus documents. All documents share the same four components and require them to be met for at least 12 months, differing in the thresholds applied. Entries refer to remission on treatment.

Consensus	Symptoms	Lung Function	CCS	Duration	Notes
Menzies-Gow 2020 (international expert) [6]	ACQ < 0.75 or ACT ≥ 20	Optimized/stable	No OCS	≥12 months	Founding framework; “complete” adds inflammatory markers
Blaiss 2023 (ACAAI/AAAAI/ATS) [8]	ACT > 20, ACQ < 0.75, no exacerbations	Stable, optimized	On or off treatment	≥12 months	Adds absence from school/ work absenteeism
Canonica 2023 (SANI, Italy) [7]	ACT ≥ 20, ACQ < 1.5, no exacerbations	Stable	No OCS	≥12 months	Less stringent symptom threshold (ACQ 1.5)
Álvarez-Gutiérrez 2024 (REMAS, Spain) [18]	No rescue needed, no exacerbations	FEV1 ≥80%, negative bronchodilator test	No systemic steroids	≥12 months	“Complete” at ≥3 years adds FeNO < 40 ppb, sputum eosinophils < 2%
Nagase 2026 (JGL, Japan) [9]	ACT ≥ 23, ACQ ≤ 0.75, no exacerbations	Optimization: normalization where achievable, otherwise stabilization	No OCS	≥12 months	Highest ACT threshold (23); lung function not tied to a numeric cut-off

AAAAI, American Academy of Allergy, Asthma and Immunology; ACAAI, American College of Allergy, Asthma and Immunology; ACQ, Asthma Control Questionnaire; ACT, Asthma Control Test; ATS, American Thoracic Society; FeNO, fractional exhaled nitric oxide; FEV1, forced expiratory volume in 1 s; JGL, Japanese asthma prevention and management guidelines; CCS, corticosteroids; OCS, oral corticosteroids; ppb, parts per billion; REMAS, Remission in Asthma; SANI, Severe Asthma Network Italy.

**Table 3:** Remission of asthma with biologic therapies: evidence from post hoc analyses of trials, real-world cohorts and registries. Studies are grouped by the cascade position of the agent evaluated, with studies covering more than one class listed separately. Rates are not comparable across studies because each used a different definition; therefore, we report them alongside every rate. The definition column provides, for each study, the depth label, the required criteria, and the endpoints used to assess them, as reported by the authors in abbreviated form. Several studies use the word “complete” for the four clinical and functional components without any biomarker (complete clinical remission), while others reserve it for definitions that add biomarker normalization, and the two usages are distinguished in that column.

Study	Design & Cohort	Biologic	Remission Definition	Remission Rate	Comorbid CRSwNP: Reported Effect on Asthma Remission
**Anti-IgE**					
Cilli 2024 [30]	Retrospective cohort, *n* = 58	Omalizumab	Clinical, 4-comp.: 0 exac., no OCS, ACQ ≤ 1 or ACT ≥ 20, FEV1 ≥ 80%	25.9% at 1–2 y; 47.4% at 5 y	Not assessed
**Anti-IL-5/IL-5Rα**					
Maglio 2023 [31]	Retrospective, multicentre, *n* = 113	Mepolizumab; benralizumab	Clinical, 4-comp. (called complete by the authors): ACQ ≤ 1.5, no SCS, FEV1 ≥ 80% or ≥LLN or within 100 mL of BL	Benralizumab 40%; mepolizumab 30.1% at 1 y	Frequent comorbidity, effect not formally tested
Bagnasco 2024 [32]	Retrospective, multicentre, *n* = 71	Mepolizumab	Clinical, SANI complete: 0 exac., no OCS, ACT ≥ 20, stable FEV1 over 12 mo (FEV1 ≥ 80% as alternative criterion)	52% at 1 y; 73% at 3 y (39% and 51% with FEV1 ≥ 80% criterion)	Assessed, not predictive of remission
Quarato 2025 [33]	Retrospective cohort, *n* = 23	Benralizumab	Clinical, SANI: complete = 0 exac., no OCS, ACT ≥ 20, stable FEV1; partial = first two plus one of the other two	Clinical 86.9% at 1 y and 91.3% at 4 y; complete 65% and 71.4%	Predicts remission
Menzies-Gow 2022 [34]	Pooled post hoc, SIROCCO/CALIMA, *n* = 1123	Benralizumab	Clinical, 3-comp.: 0 exac., no OCS, ACQ-6 ≤ 0.75; 4-comp. adds pre-BD FEV1 gain ≥100 mL	39.2% and 33.4% in the same dataset	Not assessed
D’Amato 2022 [35]	Post hoc, ANANKE study, *n* = 110 with CRSwNP	Benralizumab	Clinical, multidomain: exac., OCS use, ACT and FEV1 assessed by domain, no composite rate	Not reported as a single rate	Lower exacerbation risk and greater OCS reduction, remission not assessed
**Anti-IL-4Rα**					
Pavord 2025 [36]	Pooled post hoc, QUEST/TRAVERSE, *n* = 58	Dupilumab	Clinical, 4-comp., on treatment: 0 severe exac., no OCS, ACQ-5 < 1.5, pre-BD FEV1 stable (decline ≤5%) or improved	37.2% at 1 y (placebo 22.2%); 42.8% at 2 y; 29.5% at both	Not assessed
Tajiri 2025 [37]	Post hoc, single centre, *n* = 28	Dupilumab	Clinical 3-comp. (no symptoms, no OCS, 0 exac.); 4-comp. adds stable or optimised FEV1; complete adds resolved inflammation and negative AHR	68%; 57%; 7% at 2 y	Predicts 4-component remission
Menzella 2026 [38]	Retrospective, single centre, *n* = 28	Dupilumab	Clinical, SANI: 0 exac., no OCS, ACT ≥ 20, stable FEV1; sinonasal remission by EUFOREA/EPOS criteria	85.7% at 1 y; 100% at 2 y	Co-treated: sinonasal remission 57% at 1 year and 88% at 2 years
**Anti-TSLP**					
Gates 2025 [26]	Retrospective cohort, *n* = 175	Tezepelumab	Clinical: 0 exac., no OCS, ACQ-6 < 1.5, stable FEV1. Biological: BEC < 300/µL and FeNO < 25 ppb	Clinical 36% (T2-high 55%, T2-low 19%); biological 38%; both 15%	Not assessed
Wechsler 2024 [39]	Pooled post hoc, NAVIGATOR/ DESTINATION, *n* = 528	Tezepelumab	Clinical, on treatment: ACQ-6 ≤ 1.5, pre-BD FEV1 > 95% of BL, 0 exac., no OCS (weeks 0–52 and 52–104)	28.5% at 1 y (placebo 21.9%); 33.5% at 2 y	Not assessed
Kan-O 2026 [17]	Prospective, single-arm, TERESA, *n* = 107	Tezepelumab	Clinical: 0 exac., no OCS, ACQ-6 ≤ 1.5, stabilised FEV1 (>95% of BL). Complete: adds BEC < 150/µL and FeNO < 25 ppb	Clinical 34.6%; complete 9.3% at 1 y	Not assessed
**Multiple biologic classes**					
Thomas 2024 [40]	Two prospective registries, *n* = 453	Omalizumab; mepolizumab	Clinical, 4-comp.: 0 exac. and no OCS in previous 6 mo, ACQ-5 ≤ 1, FEV1 optimised (≥80%) or stabilised (decline ≤5%)	Mepolizumab 25.2%; omalizumab 19.1% at 1 y	Not assessed
Sposato 2023 [41]	Retrospective cohort, *n* = 486	Omalizumab; mepolizumab; benralizumab; dupilumab	Clinical, 4-comp.: ACT ≥ 20, 0 exac., no OCS, FEV1 ≥ 80% after ≥ 12 mo	Benralizumab 35.8%; mepolizumab 23.6%; dupilumab 23.5%; omalizumab 21.8% at 1 y	Predicts remission with benralizumab and mepolizumab
**Registry-level syntheses**					
McDowell 2023 [42]	UK registry, *n* = 830	Various	Clinical, 3- and 4-comp.; primary: ACQ-5 < 1.5, 0 exac., no maintenance OCS > 5 mg, FEV1 ≥ LLN or within 100 mL of BL	18.3% at 1 y (12–21% depending on the definition)	Associated with remission
McDowell 2025 [27]	UK registry, *n* = 778 adults with severe asthma	Various	Biological only: FeNO < 20 ppb and BEC < 150/µL at 1 y, no clinical criteria	19%	Not assessed
Chipps 2025 [43]	US registry, CHRONICLE, *n* = 611	Various	Clinical, on treatment: 0 exac., no SCS over 12 mo, ACT ≥ 20 (or ≥50% gain) in latest 6 mo, specialist-reported control, no FEV1 criterion	22.3% at 12–13 mo; 34.3% at 47–48 mo	Not assessed
Lee 2025 [16]	Korean registry, KoSAR, *n* = 405	Various, not exclusively biologic-treated	Clinical (CR): ACT ≥ 20, 0 exac., no OCS. Complete clinical (CCR): adds FEV1 ≥ 80% or gain ≥100 mL. No biomarker	Clinical 18.3%; complete 5.9% at 1 y	Not assessed
Perez-de-Llano 2024 [44]	International registry, ISAR, 23 countries	Various	Clinical, 2- to 4-domain: 0 exac. per y, no LTOCS, partly or well-controlled asthma, FEV1 ≥ 80%	3-domain 33.5%; 4-domain 20.3% at 1 y	Not assessed

Abbreviations: 3-component remission, absence of symptoms, absence of exacerbations and no oral corticosteroids; 4-component remission, the same plus normalised or stabilised lung function; complete remission, the four components plus normalisation of type 2 biomarkers and, in some definitions, a negative bronchial hyperresponsiveness test. ACQ, Asthma Control Questionnaire; ACT, Asthma Control Test; AHR, airway hyperresponsiveness; BEC, blood eosinophil count (cells/µL); BL, baseline; comp., component(s); CRSwNP, chronic rhinosinusitis with nasal polyps; EPOS, European Position Paper on Rhinosinusitis and Nasal Polyps; EUFOREA, European Forum for Research and Education in Allergy and Airway Diseases; exac., exacerbation(s); FeNO, fractional exhaled nitric oxide (parts per billion); FEV1, forced expiratory volume in 1 s; ISAR, International Severe Asthma Registry; KoSAR, Korean Severe Asthma Registry; LLN, lower limit of normal; mo, months; OCS, oral corticosteroids; ppb, parts per billion; pre-BD, pre-bronchodilator; SCS, systemic corticosteroids; LTOCS, long-term oral corticosteroids; T2, type 2; SANI, Severe Asthma Network Italy; y, year(s).

## Data Availability

No new data were created or analyzed in this study. Data sharing is not applicable to this article.
